# Identification and validation of the common pathogenesis and hub biomarkers in Hirschsprung disease complicated with Crohn’s disease

**DOI:** 10.3389/fimmu.2022.961217

**Published:** 2022-09-28

**Authors:** Jing Wang, Zejian Li, Jun Xiao, Luyao Wu, Ke Chen, Tianqi Zhu, Chenzhao Feng, Didi Zhuansun, Xinyao Meng, Jiexiong Feng

**Affiliations:** ^1^ Department of Pediatric Surgery, Tongji Hospital, Tongji Medical College, Huazhong University of Science and Technology, Wuhan, China; ^2^ Hubei Clinical Center of Hirschsprung Disease and Allied Disorders, Wuhan, China

**Keywords:** Crohn’s disease, Hirschsprung disease, hub genes, diagnostic model, CXCL10

## Abstract

**Background:**

Although increasing evidence has supported that Hirschsprung disease (HSCR) is the risk factor for children developing Crohn’s disease (CD), the common mechanism of its co-occurrence remains unknown. The purpose of this study is to further explore the underlying mechanism and biomarkers for the co-occurrence of HSCR and CD.

**Methods:**

The Gene Expression Omnibus (GEO) database was used to obtain gene expression profiles for CD (GSE95095) and HSCR (GSE98502). Following the identification of the shared differentially expressed genes (DEGs) of CD and HSCR, functional annotation, protein–protein interaction (PPI) network creation, and module assembly were performed to discover hub genes. RT-qPCR was performed to validate the expression of the hub genes in HSCR samples. The receiver operating characteristic (ROC) curve was utilized to assess the accuracy of the hub genes as biomarkers in predicting CD in both the training dataset and test dataset.

**Results:**

A total of 103 common DEGs (50 downregulated genes and 53 upregulated genes) were chosen for further investigation. The importance of chemokines and cytokines in these two disorders is highlighted by functional analysis. MCODE plug identified three important modules, which functionally enriched the immune system process. Finally, nine hub genes were identified using cytoHubba, including IL1B, IL10, CXCL10, ICAM1, EGR1, FCGR3A, S100A12, S100A9, and FPR1. The nine hub genes were mainly enriched in immune- and inflammation-related pathways. External data profiles and RT-qPCR confirmed the expression of the nine hub genes in HSCR and CD. ROC analysis revealed that the nine hub genes had a strong diagnostic value.

**Conclusion:**

Our study reveals the common pathogenesis of HSCR and CD. These hub genes and diagnostic models may provide novel insight for the diagnosis and treatment of HSCR complicated with CD.

## Introduction

Hirschsprung disease (HSCR) is a common congenital malformation of the enteric nervous system in children, with an incidence of approximately 1 in 5,000 births (1). HSCR is distinguished by the lack of ganglion cells in the distal colon, and removal of the aganglionic colon section is an effective therapy (2). Hirschsprung-associated enterocolitis (HAEC) is a prevalent cause of morbidity in HSCR patients and can occur at any stage of the disease’s course (3). The reported incidence of HAEC before surgery ranges from 6% to 50%, whereas it ranges from 2% to 35% after surgery (4). The pathophysiology of HAEC is complex, and our knowledge of this inflammatory disease is still limited (5).

Inflammatory bowel disease (IBD), which includes Crohn’s disease (CD) and ulcerative colitis, is caused by the combination of environmental and genetic variables in the gastrointestinal system, resulting in immune responses and inflammation (6). The increasing global burden of IBD brings critical challenges to healthcare systems all over the world as they work to care for this complex and costly disease (7, 8). According to estimates, over 1.4 million Americans and several million people worldwide were diagnosed with IBD in 2005, with 30% of them being adolescents and young adults aged 10 to 30 years (9). In recent years, more and more studies have shown that the risk of IBD, especially CD, increased in children with HSCR (10–12). Sherman et al. reported on nine individuals with HSCR and IBD in early 1989 and proposed a link between the two illnesses (13). Importantly, HAEC and IBD share similar clinical presentations such as diarrhea, abdominal distension, and colicky abdominal pain (14). The exact pathogenesis association of HSCR and IBD is not known. However, both HSCR and IBD are characterized by an impaired intestinal mucosal barrier function, which may be a common pathway. The common transcription feature may provide novel insights into the common pathogenesis of HSCR and CD. The goal of this study is to find hub genes involved in the pathogenesis of HSCR complicated with CD. The HSCR and CD colon tissue expression profiles were obtained from the Gene Expression Omnibus (GEO) database for integration analysis. Comprehensive bioinformatics and enrichment analysis were performed to uncover the common differentially expressed genes (DEGs) and their functions of HSCR and CD. Furthermore, using the STRING database and the Cytoscape program, a protein–protein interaction (PPI) network was built to examine gene modules and discover hub genes. In the end, we identified nine hub genes, including CXCL10, EGR1, ICAM1, IL10, IL1B, S100A9, S100A12, FCGR3A, and FPR1. We investigated the underlying mechanism of these genes and confirmed their expression in external datasets and HSCR tissues. Lastly, the receiver operating characteristic (ROC) curve was used to assess the accuracy of the hub markers in diagnosing CD. The hub genes and diagnostic model identified between HSCR and CD are expected to provide new insights into the underlying mechanisms of the co-occurrence of HSCR and CD.

## Materials and methods

### Data source

Related gene expression datasets were searched using Hirschsprung disease and inflammatory bowel disease as keywords. Two microarray datasets GSE98502 and GSE95095 were downloaded from the GEO database (http://www.ncbi.nlm.nih.gov/geo). The GSE98502 dataset contains colon tissue from eight HSCR patients and eight normal controls. The GSE95095 dataset consists of 48 CD patients and 12 normal healthy controls. Moreover, the validation datasets GSE126124 (CD), GSE117993 (CD), and GSE96854 (HSCR) were also downloaded from the GEO database.

### Identification of differentially expressed genes

Raw data were read using two R packages (“GEOquery” and “Limma”), and the Limma package was used to generate the differential expression. The gene expression profiles of different groups were compared using “Limma” to determine the DEGs between the case and control groups. Probe sets that did not have a corresponding gene symbol were eliminated. Genes with multiple probe sets were averaged. The genes with p-value <0.01 and |fold change| ≥ 1 were identified as DEGs. The Venn diagram was used to obtain the common DEGs between HSCR and CD datasets.

### Enrichment analyses of differentially expressed genes

Gene Ontology (GO) is an established international classification system for gene function that provides a dynamically updated regulated vocabulary and rigorously defined concepts to identify genes and their products thoroughly (15). GO has three ontologies: molecular functions, cellular components, and biological processes. GO enrichment analysis provides all GO terms that are significantly enriched for DEGs compared to genomic background and filters for DEGs corresponding to biological functions. Kyoto Encyclopedia of Genes and Genomes (KEGG) is the main public pathway-related database. Pathway enrichment analysis identifies pathways that are significantly enriched in DEGs compared to the genome-wide background (16). GO and KEGG enrichment analyses were performed with the “clusterProfiler” R package.

### Protein–protein interaction network construction and module analysis

The online search tool STRING was used to explore the interacting Genes (http://string-db.org) (17). To build a PPI network with multiple regulatory linkages, the STRING database allows the search for relationships between proteins of interest, such as direct binding relationships or coexisting upstream and downstream regulatory pathways. Interactions with a combined score over 0.4 were considered statistically significant. Cytoscape (http://www.cytoscape.org) software (18) was used to visualize the PPI network. The following selection criteria were utilized to examine major functional modules using Cytoscape’s plug-in molecular complex detection technology (MCODE): K-core = 2, degree cutoff = 2, max depth = 100, and node score cutoff = 0.2. The KEGG and GO analyses of the key modular genes were performed.

### Selection and analysis of hub genes

The hub genes were discovered using Cytoscape’s cytoHubba plug-in. To analyze the hub genes, seven standard algorithms (MCC, MNC, Degree, Closeness, Radiality, Stress, and EPC) were employed. The shared key hub genes across these seven approaches were filtered using an upset diagram. Subsequently, a co-expression network of these hub genes was established *via* GeneMANIA (http://www.genemania.org/) (19), which is a widely used tool revealing the internal associations in gene sets.

### Validation of hub gene expression in other datasets and Hirschsprung disease samples

The mRNA expression of identified hub genes was verified in both GSE124126 and GSE117993. The GSE124126 dataset contains 21 controls and 37 CD patients. GSE117993 consists of 55 controls and 32 CD samples. Both GSE124126 and GSE117993 samples were the human bowel tissue in CD. A three versus three HSCR samples dataset (GSE96854) was also used for validation. The comparison between the two sets of data was performed with the t-test. p-Value <0.05 was considered significant. RT-qPCR was performed to analyze the mRNA expression of hub genes in HSCR samples and normal controls. This study complied with the Declaration of Helsinki and was approved by the Review Board of the Ethics Committee of Tongji Hospital. Consent forms were sent and signed by their legal custodians. The Institutional Review Board of Tongji Hospital, Tongji Medical College, Huazhong University of Science and Technology approved the protocol of the study. Total RNA was isolated from colon tissues with TRIzol reagent (Life Technologies, Carlsbad, CA, USA). qPCR and data analysis were performed using LightCycler96 (Roche Diagnostics, Basel, Switzerland). The levels of relative expression were estimated using β-actin as an internal reference. The experiments were conducted three times with biological duplicates. Full-length resected bowel specimens were obtained during pull-through operations of HSCR patients (n = 10). Aganglionic and ganglionic segments were resected. Ganglionic segments were collected from the resected pull-through specimen’s most proximal margin, whereas aganglionic segments were taken from the resected specimen’s most distal margin. A control group of specimens was taken from imperforate anus patients following colostomy (n = 10).

### Diagnostic value of hub genes in Crohn’s disease

ROC was plotted using the pROC package to assess the performance of hub genes distinguishing between CD and healthy individuals. Logistics regression analysis was used to establish an integrated diagnostic model using the nine hub genes. In addition, the diagnostic value of the hub genes and the integrated diagnostic model was validated with a separate external dataset.

### Immunofluorescence staining and confocal microscopy

HSCR-CD (n = 3), HSCR (n = 10), and normal (n = 10) colon tissue sections were performed with immunofluorescence staining using the anti-CXCL10 antibody (A1457, ABclonal, Woburn, MA, USA) as previously described (20). All sections were independently evaluated by two researchers with LSM 800 confocal microscope (Carl Zeiss MicroImaging GmbH, Jena, Germany).

### Statistical analysis

The embryos were selected by Simple random sampling. Data were analyzed using the GraphPad Prism software and presented as the mean ± SD. Differences between the two groups were analyzed using an unpaired t-test with Welch’s correction. Analysis of variance (ANOVA) was used to compare data from more than two groups. p < 0.05 was considered statistically significant.

## Results

### Identification of differentially expressed genes in Hirschsprung disease and Crohn’s disease expression profiles

**Figure 1:**
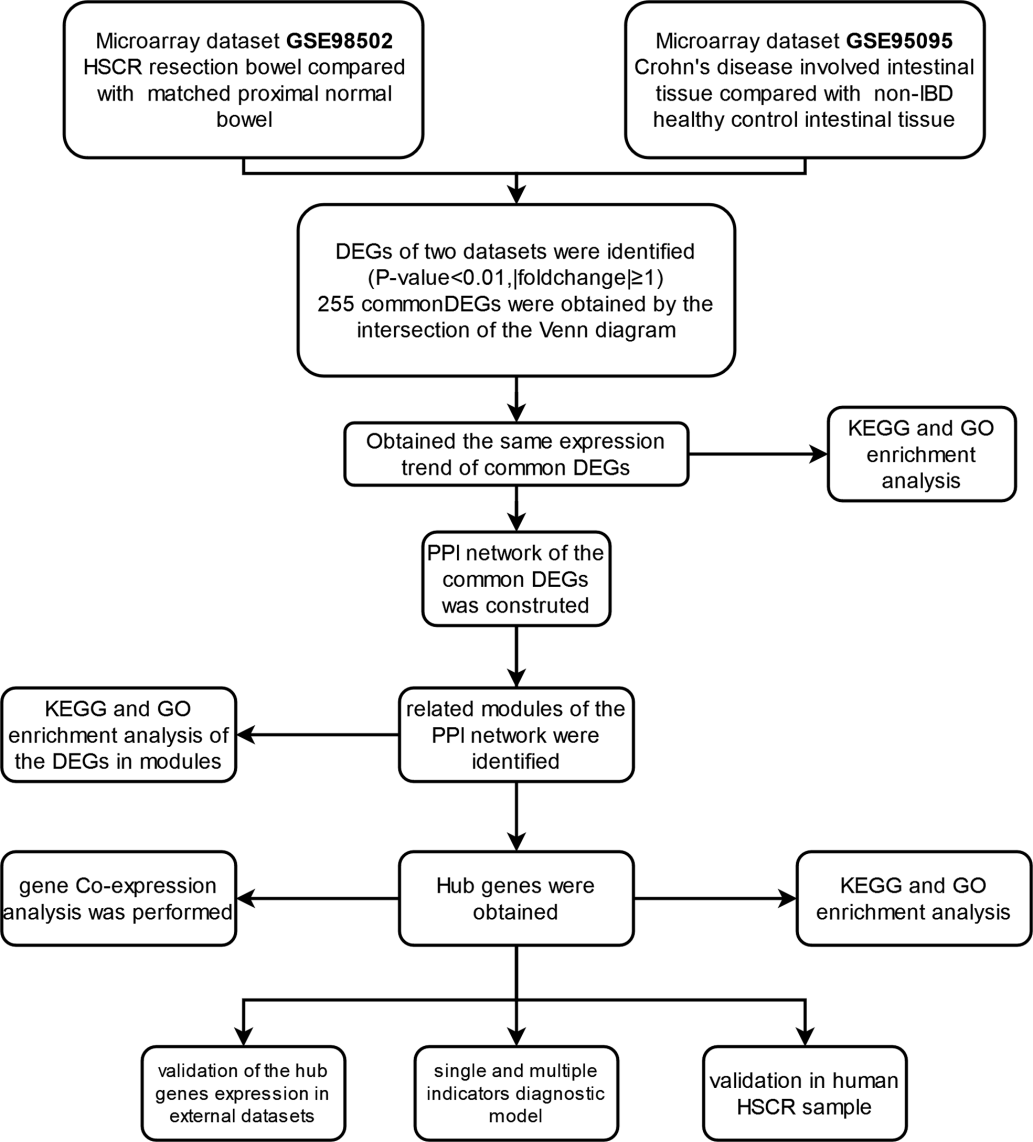
Research design flowchart.

**Figure 2:**
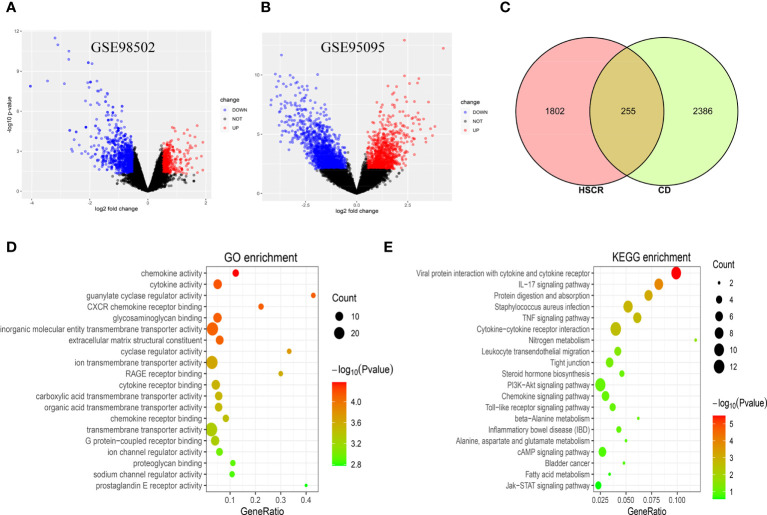
**(A)** The volcano map of GSE98502. **(B)** The volcano map of GSE95095. Upregulated genes are marked in red; downregulated genes are marked in blue. **(C)** The two datasets showed an overlap of 255 DEGs. **(D, E)** The enrichment analysis results of GO and KEGG pathway. DEGs, differentially expressed genes; GO, Gene Ontology; KEGG, Kyoto Encyclopedia of Genes and Genomes.

The research flowchart of this study is shown in **Figure 1**. After normalizing the microarray results, DEGs (2,057 in GSE98502 and 2,641 in GSE95095) were identified (**Figures 2A, B**). A total of 255 common DEGs were identified by taking the intersection of the Venn diagram (**Figure 2C**). Finally, 103 DEGs were obtained with the same expression trends, including 53 upregulating genes and 50 downregulating genes.

### Analysis of the functional characteristics of common differentially expressed genes

The GO and KEGG pathway enrichment analyses were used to investigate the biological activities and pathways involved in the 103 common DEGs. GO analysis results show that these genes were mainly enriched in chemokine activity (p = 4.23E−05), cytokine activity (p = 6.83E−05), guanylate cyclase regulator activity (p = 7.55E−05), and CXCR chemokine receptor binding (p = 7.71E−05) (**Figure 2D**). In terms of the KEGG pathway, the three significant enrichment pathways are Viral protein interaction with cytokine and cytokine receptor (p = 3.32E−06), IL-17 signaling pathway (p = 0.00012), and Protein digestion and absorption (p = 0.00074) (**Figure 2E**). These findings strongly suggest that chemokines and cytokines are both implicated in the onset and progression of these two inflammatory disorders.

### Protein–protein interaction network construction and module analysis

**Figure 3:**
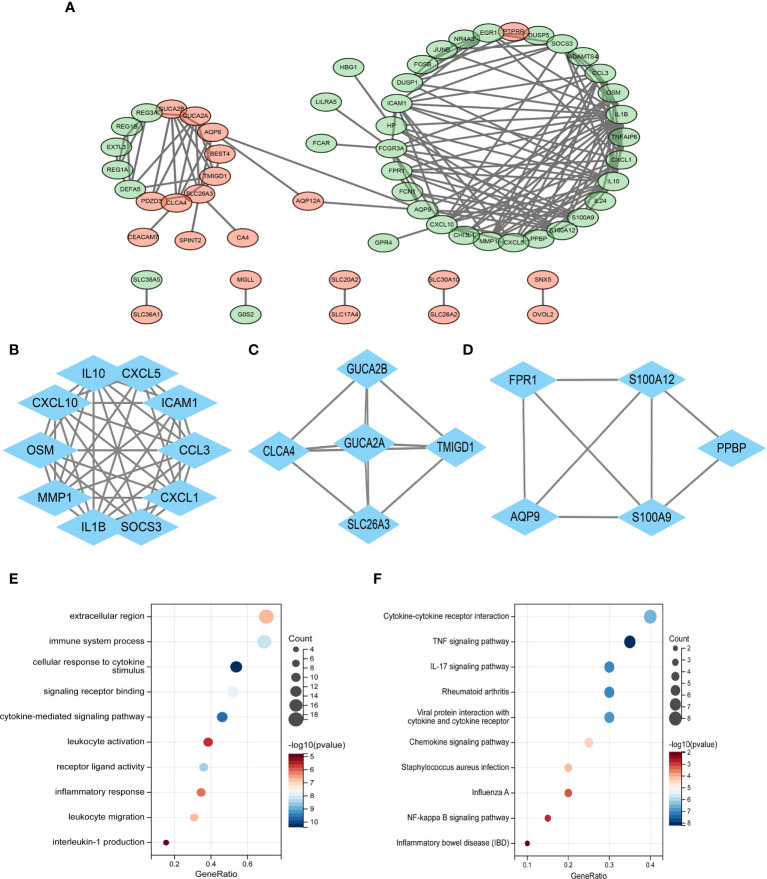
**(A)** PPI network diagram. Green indicates upregulated genes, and red-violet indicates downregulated genes. **(B–D)** Three significant gene clustering modules. **(E–F)** GO and KEGG enrichment analyses of the modular genes. The size of the circle represents the number of genes involved, and the abscissa represents the frequency of the genes involved in the term total genes. PPI, protein–protein interaction; GO, Gene Ontology; KEGG, Kyoto Encyclopedia of Genes and Genomes.

With the use of Cytoscape, a PPI network of common DEGs with total scores larger than 0.4 was created, comprising 60 nodes and 160 interaction pairings (**Figure 3A**). The MCODE plug-in of Cytoscape was used to obtain the most closely related gene modules, which included 20 common DEGs and 59 interaction pairings (**Figures 3B–D**). GO analysis showed that these genes are related to the extracellular region, immune system response, and cellular response to cytokine stimulus (**Figure 3E**). KEGG pathway analysis revealed that they are mostly involved in cytokine–cytokine receptor interaction, TNF signaling pathway, IL-17 signaling pathway, and IBD (**Figure 3F**).

### Selection and analysis of hub genes

**Figure 4:**
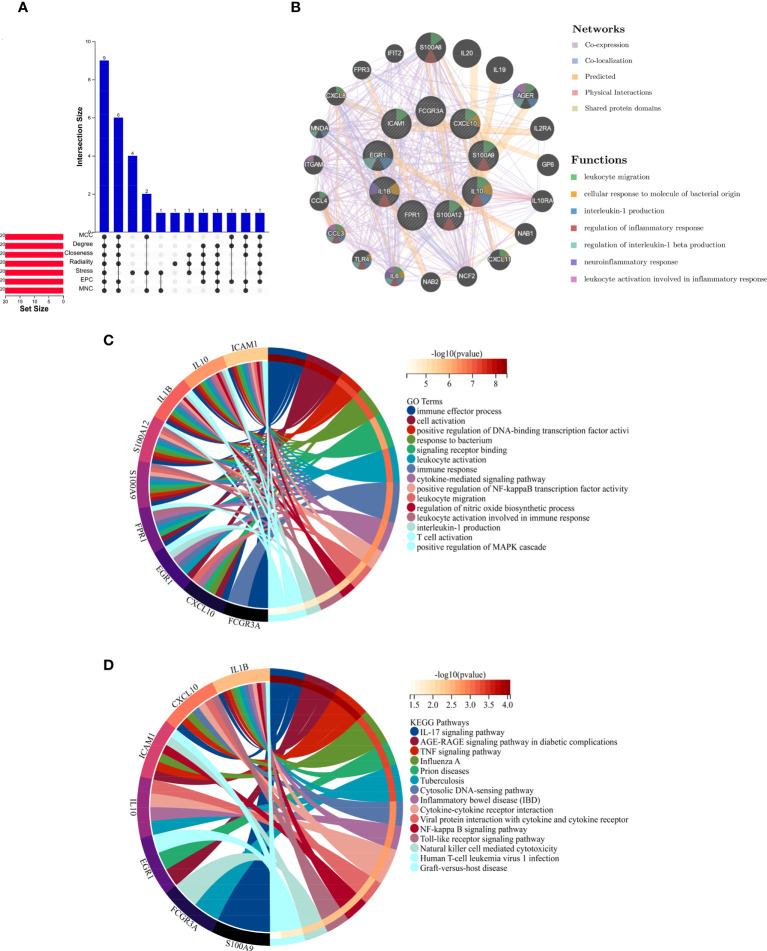
**(A)** The Venn diagram shows that seven algorithms have screened out nine overlapping hub genes. **(B)** Hub genes and their co-expression genes were analyzed *via* GeneMANIA. **(C, D)** GO and KEGG enrichment analyses of the hub genes. The outermost circle is term on the right, and the inner circle on the left represents the significant p-value of the corresponding pathway of the gene. GO, Gene Ontology; KEGG, Kyoto Encyclopedia of Genes and Genomes.

The top 20 hub genes were calculated using the seven algorithms of the cytoHubba plug-in (**Table 1**). After taking the intersection of the Venn diagrams, nine common hub genes, including IL1B, IL10, CXCL10, ICAM1, EGR1, FCGR3A, S100A12, S100A9, and FPR1, were found (**Figure 4A**). **Table 2** lists their entire names as well as the functions they serve. The co-expression network and associated functions of these hub genes were investigated using the GeneMANIA database. These hub genes showed the complex PPI network with a co-expression of 77.09%, physical interactions of 4.53%, co-localization of 11.69%, prediction of 5.88%, and share protein of domains of 0.99% (**Figure 4B**). GO analysis showed that these genes are mainly involved in the immune effector process, cell activation, immune response, and cytokine-mediated signaling pathway (**Figure 4C**). In addition, KEGG pathway analysis showed that they are mainly involved in the IL-17 signaling pathway and TNF signaling pathway. Interestingly, two hub genes (IL1B and IL10) were also involved in IBD (**Figure 4D**).

**Table 1:** Top 20 hub genes in seven algorithms.

MCC	Degree	Closeness	Radiality	Stress	EPC	MNC
IL1B	CCL3	IL1B	IL1B	AQP9	CCL3	IL1B
IL10	ICAM1	IL10	AQP9	AQP8	ICAM1	IL10
CXCL10	CXCL10	FCGR3A	FCGR3A	IL1B	CXCL10	CXCL10
CCL3	CXCL1	CXCL10	S100A12	GUCA2A	CXCL1	CXCL1
CXCL1	IL10	CXCL1	S100A9	GUCA2B	IL10	CCL3
ICAM1	IL1B	ICAM1	FPR1	FCGR3A	IL1B	ICAM1
MMP1	MMP1	AQP9	IL10	REG3A	MMP1	OSM
CXCL5	FCGR3A	S100A12	AQP8	SLC26A3	FCGR3A	SOCS3
OSM	SOCS3	S100A9	ICAM1	EGR1	SOCS3	MMP1
SOCS3	OSM	CCL3	CXCL1	S100A12	OSM	EGR1
FCGR3A	CXCL5	SOCS3	CXCL10	S100A9	CXCL5	FCGR3A
S100A9	S100A12	FPR1	SOCS3	IL10	S100A12	S100A12
FPR1	S100A9	OSM	CCL3	CXCL10	S100A9	S100A9
S100A12	FPR1	EGR1	EGR1	DUSP1	FPR1	GUCA2A
PPBP	PPBP	MMP1	OSM	HP	PPBP	FPR1
AQP9	EGR1	DUSP1	MMP1	FPR1	EGR1	CXCL5
CHI3L1	AQP9	CXCL5	DUSP1	DEFA5	AQP9	GUCA2B
EGR1	DUSP1	PPBP	JUNB	CLCA4	DUSP1	PPBP
GUCA2A	JUNB	AQP8	AQP12A	JUNB	JUNB	SLC26A3
GUCA2B	CHI3L1	JUNB	CXCL5	ICAM1	CHI3L1	JUNB

**Table 2:** The detail of hub genes.

No.	Gene symbol	Full name	Function
1	IL1B	Interleukin 1 beta	This cytokine is an important mediator of the inflammatory response and is involved in a variety of cellular activities, including cell proliferation, differentiation, and apoptosis.
2	IL10	Interleukin 10	This cytokine has pleiotropic effects in immunoregulation and inflammation. This cytokine can block NF-kappa B activity and is involved in the regulation of the JAK-STAT signaling pathway.
3	CXCL10	C-X-C motif chemokine ligand 10	This cytokine has pleiotropic effects, including stimulation of monocytes, natural killer and T-cell migration, and modulation of adhesion molecule expression.
4	ICAM1	Intercellular adhesion molecule 1	This gene encodes a cell surface glycoprotein, which is typically expressed on endothelial cells and cells of the immune system.
5	EGR1	Early growth response 1	The protein encoded by this gene belongs to the EGR family of C2H2-type zinc-finger proteins. It is a nuclear protein and functions as a transcriptional regulator.
6	FCGR3A	Fc gamma receptor IIIa	This gene is involved in the removal of antigen–antibody complexes from the circulation, as well as other responses, including antibody-dependent cellular-mediated cytotoxicity and antibody-dependent enhancement of virus infections.
7	S100A12	S100 calcium-binding protein A12	The protein encoded by this gene is a member of the S100 family of proteins containing 2 EF-hand calcium-binding motifs. In smooth muscle cells, this protein co-expresses with other family members in the nucleus and stress fibers, suggesting diverse functions in signal transduction.
8	S100A9	S100 calcium-binding protein A9	The protein encoded by this gene is a member of the S100 family of proteins containing 2 EF-hand calcium-binding motifs. This protein may function in the inhibition of casein kinase, and altered expression of this protein is associated with the disease cystic fibrosis.
9	FPR1	Formyl peptide receptor 1	This gene encodes a G protein-coupled receptor of mammalian phagocytic cells. The protein mediates the response of phagocytic cells to invasion of the host by microorganisms and is important in host defense and inflammation

### Validation of hub gene expression

**Figure 5:**
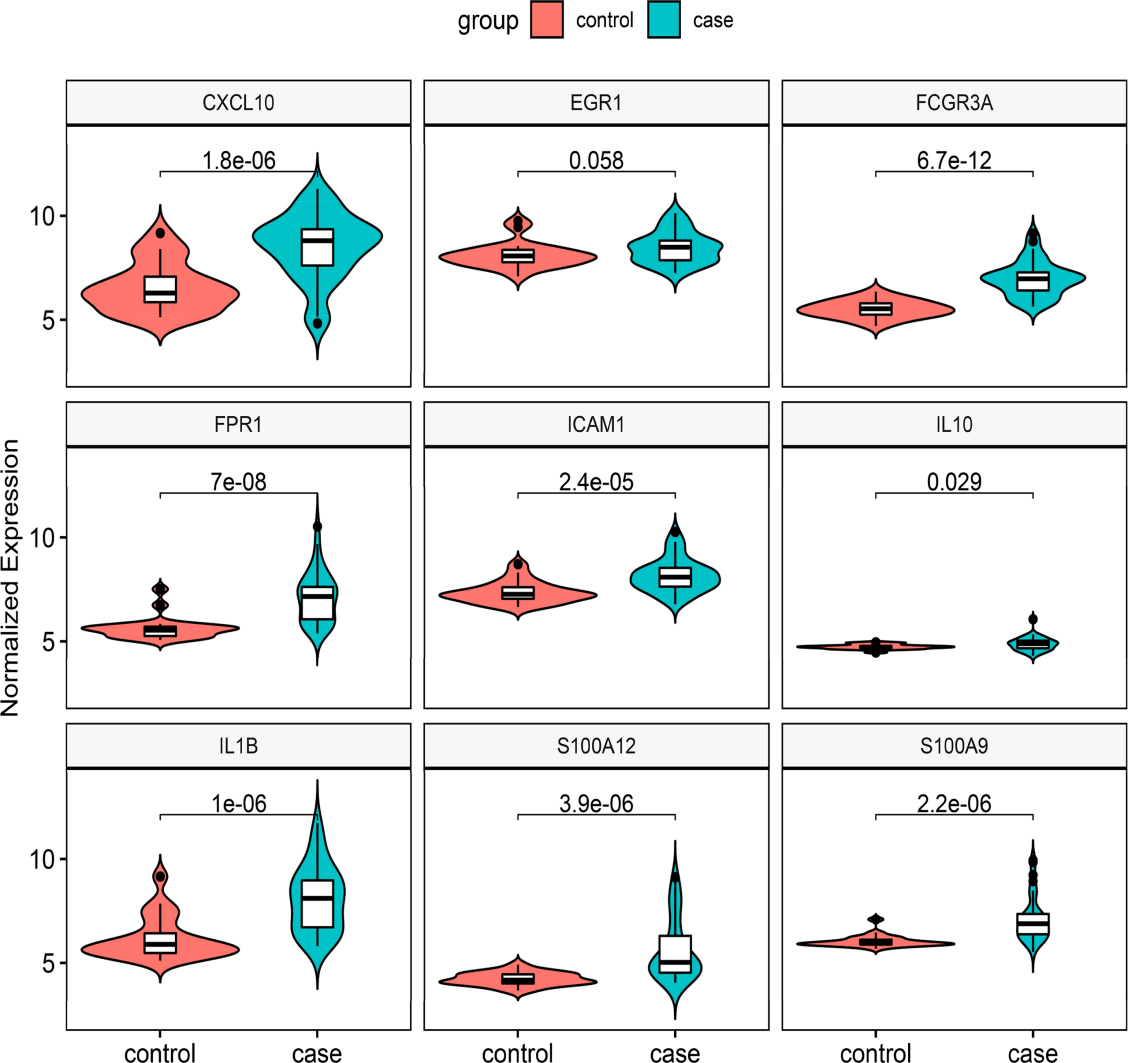
Validation of the nine hub genes’ expression in external CD gene expression profile (GSE126124). CD, Crohn’s disease.

**Figure 6:**
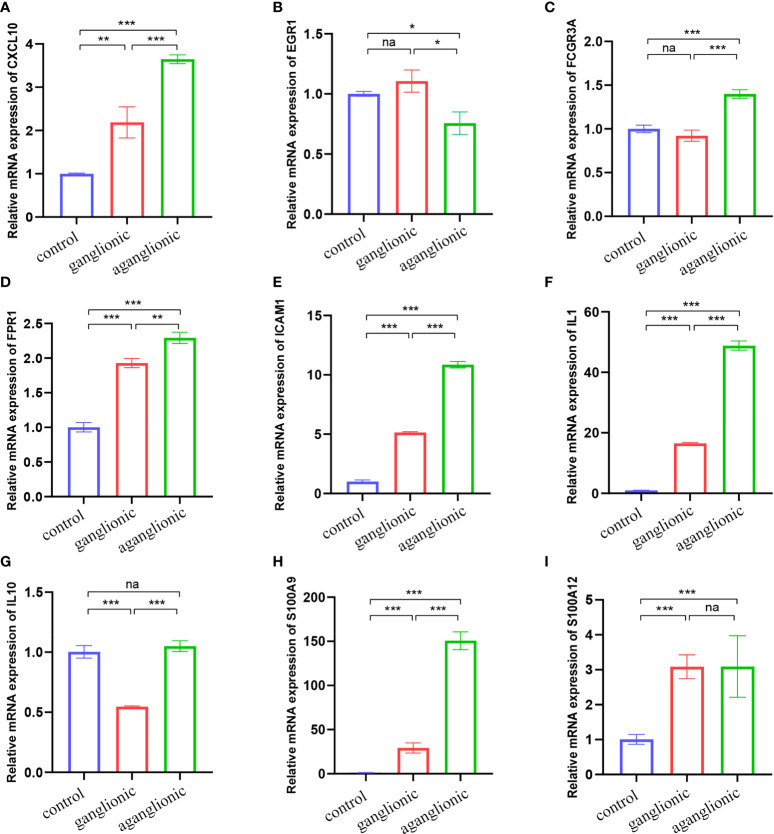
Validation of the nine hub genes’ expression in HSCR and normal colon tissue samples by RT-qPCR (n = 10). **(A)** CXCL10, **(B)** EGR1, **(C)** FCGR3A, **(D)** FPR1, **(E)** ICAM1, **(F)** IL1B, **(G)** IL10, **(H)** S100A9 and **(I)** S100A12. Na, not significant; HSCR,Hirschsprung disease. *, p < 0.05; **, p 0.01; ***, p 0.001.

We chose two more CD datasets and an HSCR dataset to validate the expression levels of these hub genes. The results showed that compared with normal bowel, all the hub genes except EGR1 were significantly upregulated in the CD-involved bowel tissue in the external dataset (GSE123126) (**Figure 5**). Similarly, the expression of all genes except EGR1 and IL10 in CD involved colon was also higher than in normal colon tissues in another dataset (GSE117993) (**Figure S1**). We also performed expression validation in another HSCR microarray dataset (GSE96854) with three HSCR colon samples and three normal colon samples (**Figure S2**). No significant results were obtained due to the small sample size. Therefore, we performed RT-qPCR to confirm the expression of the hub gene in HSCR tissue. As shown in **Figure 6**, we found that the mRNA expression of hub genes CXCL10, FCGR3A, FPR1, IL1B, and S100A9 was significantly higher in the aganglionic segment than ganglionic segment and control normal segment. The expression of EGR1 was reduced in the aganglionic segment compared to the ganglionic segment and controlled normal colon. The expression of ICAM1 in the aganglionic segment was reduced compared to the ganglionic colon and increased when compared to the normal colon. The expression level of IL10 was significantly reduced in the ganglionic segment. The expression of S100A12 was increased in both ganglionic and aganglionic segments compared to the control normal colon.

### Diagnostic value of hub genes in Hirschsprung disease–Crohn’s disease

**Figure 7:**
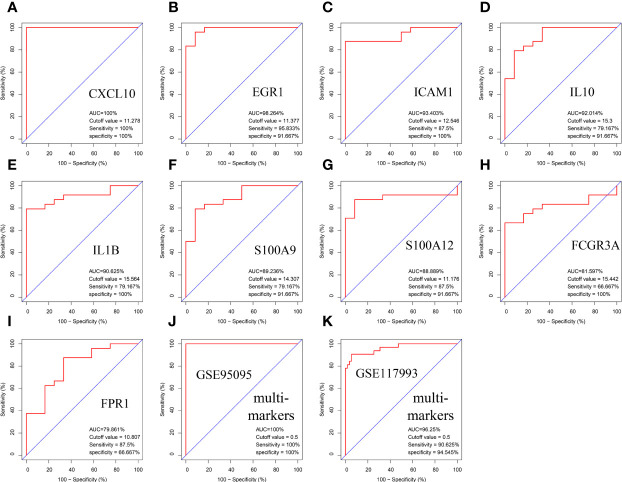
Validation of hub genes in the diagnostic value. **(A–I)** Validation of hub genes in the GSE95095. ROC curves and AUC statistics are used to evaluate the capacity to discriminate CD from healthy controls with excellent sensitivity and specificity. **(J)** Validation of the integrated diagnostic model in the GSE95095. **(K)** Validation of the integrated diagnostic model in the GSE117993. These findings indicated these nine hubgenes have excellent diagnostic efficiency in CD. ROC, receiver operating characteristic; AUC, area under the curve; CD, Crohn’s disease.

The area under the curve (AUC) values of the nine hub genes were evaluated by ROC curve analysis to examine their sensitivity and specificity for the diagnosis of CD. Eight of nine hub genes had AUC values more than 0.8, indicating that they have a strong diagnostic value for CD (**Figures 7A–I**). To further confirm their diagnosis performance, the diagnostic value of the abovementioned nine hub genes was also validated in the GSE126124 dataset (**Figure S3**). For better performance in prognosis prediction, the nine hub genes were integrated to establish a multi-marker diagnosis model by logistics regression analysis. The ROC showed that the multi-marker model could effectively predict the diagnosis of CD (AUC = 1.00) (**Figure 7J**). In addition, the multi-marker diagnostic model also performed well in the validation dataset GSE117993 (AUC = 0.96) (**Figure 7K**).

### Aberrant CXCL10 expression in Hirschsprung disease–Crohn’s disease

**Figure 8:**
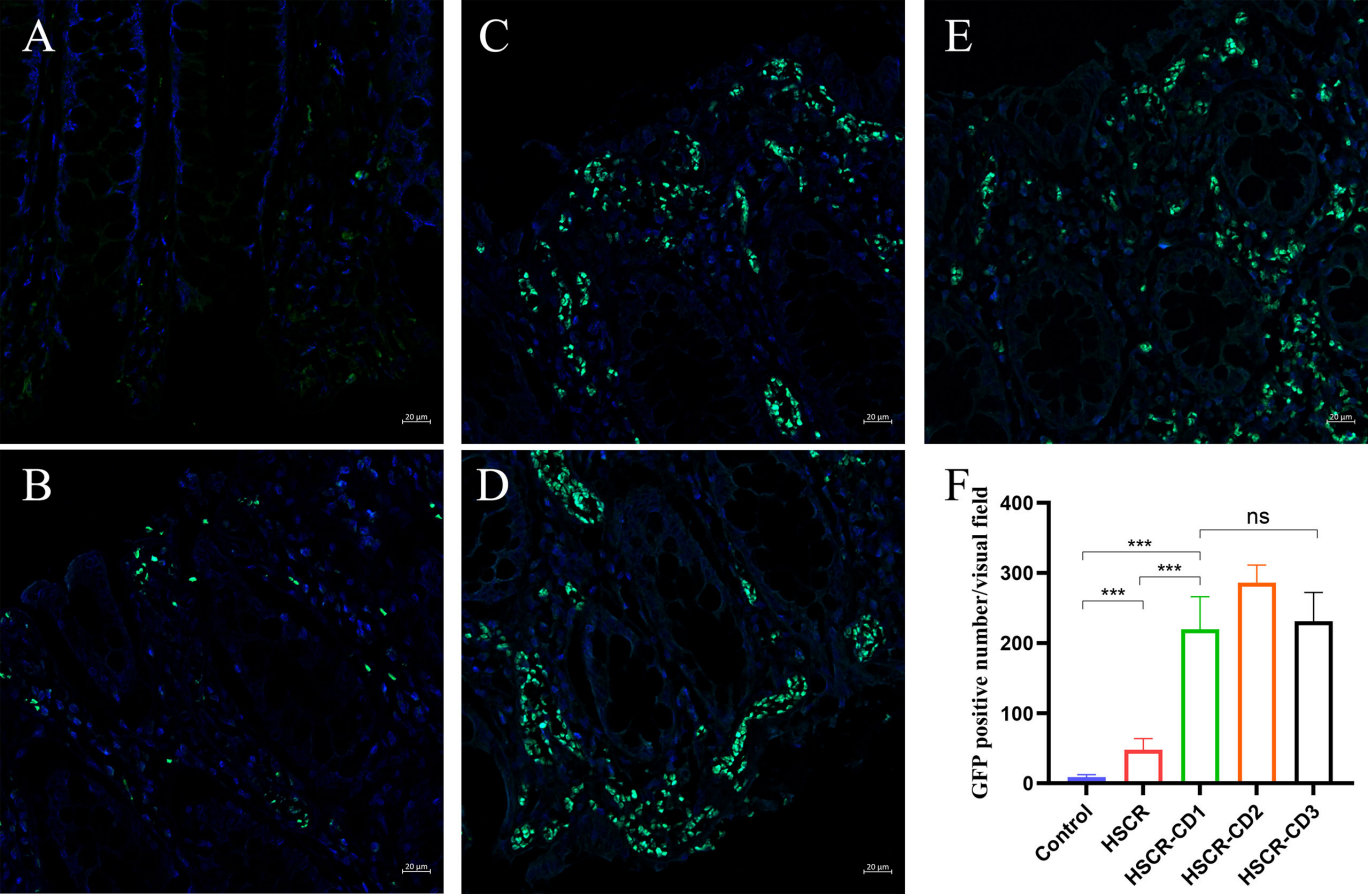
Representative immunofluorescent staining images of CXCL10 expression in control **(A)**, HSCR **(B)**, HSCR-CD patient 1 **(C)**, HSCR-CD patient 2 **(D)**, and HSCR-CD patient 3 **(E)** colon tissues. **(F)** statistical analysis of GFP-positive cell in each group. ***, p < 0.001. ns, not significant; HSCR, Hirschsprung disease; CD, Crohn’s disease.

As shown in **Figures 8A–E**, in normal healthy colon tissues, CXCL10 is essentially unexpressed or sparsely expressed. In disease conditions (HSCR and HSCR-CD), CXCL10 was expressed abundantly in the intestinal mucosa. Moreover, the statistical result (**Figure 8F**) revealed that the expression of CXCL10 was significantly increased in HSCR-CD colon tissues compared to HSCR and normal controls colon tissues (p < 0.001).

## Discussion

In this study, the pathogenic association between HSCR and CD was explored. Nine hub genes were identified, and associated mechanisms were revealed for the co-occurrence of HSCR and CD. A multi-marker diagnostic model was established and performed well in predicting the occurrence of CD.

More and more studies have reported that patients with HSCR had a greater risk of developing IBD, especially CD, than patients without HSCR. A population-based cohort research showed that the incidence of IBD in patients with HSCR was 168.8 per 100,000 person-years compared to 14.2 per 100,000 person-years in children without HSCR (11). However, the cause for the link between IBD and HSCR is unknown. Approximately 200 risk loci have been identified for IBD genetically, and more than 16 genes have been uncovered that are associated with HSCR, but there are no definite links between HSCR and IBD (21, 22). Both CD and IBD may be triggered by environmental factors, but no obvious related risk has been identified. Environmental risk factors for IBD include smoking, antibiotic use in childhood, low vitamin D levels, and diet (23). In terms of HSCR, benzophenone-3, lovastatin, and mycophenolic have been reported to cause the delayed development of the enteric nervous system and HSCR-like phenotypes (24, 25).

The purpose of this study was to explore the underlying pathogenic association and mechanisms between HSCR and CD. HAEC has been proposed as a potential relationship between HSCR and IBD. HAEC is a significant consequence of HSCR that accounts for both HSCR mortality and morbidity. HAEC is expected to occur in up to 45% of HSCR patients (26). It can be speculated that the influence of the gut microbiome plays an important role in the pathogenesis of IBD and HAEC. It has been demonstrated that patients with HAEC have an altered bacterial and fungal microbiome (27). Furthermore, patients with IBD have significantly altered gut microbiome profiles as compared to persons without IBD (28, 29). In our study, we found out that HSCR and CD might share overlapping pathogenic pathways. The GO enrichment analysis of hub genes showed that the immune effector process, leukocyte activation, immune response, and response to bacterium were enriched. The KEGG enrichment analysis of common DEGs showed that the IL-17 signaling pathway, TNF signaling pathway, cytokine–cytokine receptor interaction, and IBD were significantly enriched. We also examined the mRNA expression levels of these hub genes in CD datasets and human HSCR samples. The results showed that five hub genes, CXCL10, FCGR3A, PFR1, IL1B, and S100A9, were significantly higher in the CD group and aganglionic segment of the HSCR group. These results indicated that HSCR and CD share common pathways during the occurrence and development of HSCR complicated with CD.

Among these hub genes, CXCL10 is secreted by immune cells (T, B, NK, and myeloid) and non-immune cells (epithelial, endothelial, and fibroblast) in response to such inflammatory stimuli (30). Anti-CXCL10 Ab therapy significantly reduced local production of myeloid-derived inflammatory cytokines and intestinal tissue damage in the innate-mediated murine colitis model. Targeting CXCL10 could be an attractive approach to treating IBD (31). However, CXCL10 has not been reported in HSCR. In our results, CXCL10 was significantly increased in the aganglionic segment. What is more, our results showed first that CXCL10 was significantly increased in the colon tissues of HSCR-CD patients, which suggested that CXCL10 might be a potential clinical biomarker for the development of CD in HSCR patients. EGR1 is a transcription factor that was discovered by its distinctive expression pattern in response to growth and differentiation mediators (32). EGR1 was shown to be upregulated in CD patients’ intestinal tissues, and it affects the expression of various immune-related genes, including TNF and intercellular adhesion molecule-1 (33). Moreover, consistent with our experiment results, EGR1 was downregulated in the narrow segments of HSCR and might involve in the development of HSCR (34). The FCGR3A is expressed on macrophages and natural killer cells, and its polymorphism affects infliximab pharmacokinetics and time to relapse in CD patients (35, 36). FPR1 is abundantly expressed on neutrophils and macrophages and stimulates their migration into the mucosa and lumen in response to formyl peptides generated from resident bacteria during the development of colitis (37). FPR1 could markedly impact the macroscopic and microscopic features of CD, leading to significant upregulation of the pro-inflammatory signaling pathway in the inflamed colon (38). No studies have been reported about FCGR3A and FPR1 in HSCR, and both were also increased in the aganglionic segments of HSCR in our results. ICAM1 is a transmembrane glycoprotein that can mediate cell adhesion, chemotaxis, lymphocyte homing, and other biological activities, as well as engage in inflammation and immunological response, and play a role in physiological and pathological processes (39). In IBD intestinal tissue, ICAM1 expression and distribution are significantly increased and are intimately correlated to the degree of tissue inflammation (40). Anti-ICAM1 monoclonal antibody prophylactic treatment had been reported that could alleviate symptoms in a dextran sulfate sodium (DSS)-induced colitis model (41). Recently, a genome-wide scan analysis implicated that the ICAM1 polymorphisms may be associated with IBD (42). In HSCR, the expression of ICAM1 was increased in the aganglionic segment, and it is hypothesized that the antigen-presenting cell function of the hypertrophic nerve trunks and abnormal transitional ganglia may be mediated in part through ICAM1 (43).

It is generally recognized that IL-1β is a constituent of the combination of pro-inflammatory cytokines responsible for the inflammation seen in IBD patients and that increases in IL-1β levels are related to higher disease severity (44). Similarly, we found that the expression level of IL-1β was also increased in the aganglionic segment. In terms of IL10, IL-10 participate in a variety of inflammatory response pathways, regulating the expression of inflammatory mediators, or interacting with other inflammatory factors, such as IL-6 and IFN-γ, to jointly regulate the inflammatory response (45). IL-10 maintains intestinal mucosal barrier homeostasis by regulating intestinal epithelial cells (46). The polymorphisms of IL-10 and IL-10 receptors are strongly correlated with the expression of effectors T cells, the degree of colonic inflammation, and pathological changes in IBD (47–49). However, the specific roles of IL10 in HSCR have not been fully elucidated.

Non-invasive inflammatory indicators offer great therapeutic relevance in the treatment of IBD. Fecal biomarkers have significant benefits over other types of biomarkers (50). The S100 protein family consists of at least 21 members, all of which are calcium-binding proteins with distinct structures. S100A8, S100A9, and S100A12, members of the calgranulin subfamily, are the most important indicators of inflammatory conditions. The expression of S100A9 varies between IBD patients and healthy controls, as well as between active and inactive illnesses. As a result, S100A9 was also used as a blood-based biomarker for IBD monitoring (51). S100A12 is a disease-specific marker, and higher fecal levels of S100A12 are observed in active IBD due to the disease’s abundant infiltrating neutrophils (52). Both S100A9 and S100A12 are increased in the aganglionic segment compared to the control normal colon. However, the function of S100A9 and S100A12 in the development of HSCR has not been reported.

Childhood IBD is linked with more widespread illness, increased disease activity, and a more convoluted disease course than adult-onset IBD. The average delay from onset to diagnosis of IBD in newborns is 11 months. Predictive models that accurately diagnose the disease or the severity of the condition will help to stratify risk, guide individualized treatment, and improve the quality of life of the child (53). Although there have been many models to predict the onset of IBD, including serum marker tests, pathological tissue tests, gut microflora tests, and genomic tests, predictive models for the onset of IBD due to HSCR have not been reported. We obtained nine hub genes by integrating the expression matrices of HSCR and CD. We utilized these hub genes to build a diagnostic prediction model, which produced accurate predictions and was further examined using external data. These hub genes and diagnostic models will be beneficial to the prevention and diagnosis of postoperative concurrent CD after HSCR and provide a theoretical basis for the molecular mechanism of HSCR and CD co-occurrence.

In conclusion, a total of nine hub genes involved in the development and progression of HSCR-CD were identified and integrated into diagnostic models for CD. This study provided novel insights into the shared pathogenesis and mechanisms of the occurrence of HSCR and CD.

## Data availability statement

The original contributions presented in the study are included in the article/**Supplementary Material**. Further inquiries can be directed to the corresponding authors.

## Ethics statement

This study was reviewed and approved by The Institutional Review Board of Tongji Hospital, Tongji Medical College, Huazhong University of Science and Technology. Written informed consent to participate in this study was provided by the participants’ legal guardian/next of kin.

## Author contributions

JF and XM conceived and designed the study. XM, JW, and ZL performed the experiment and the data analysis. XM and JW wrote the original manuscript. JX, LW, KC, TQZ, CF, and DZ supervised and revised the manuscript. JF provided funding acquisition. All authors contributed to the article and approved the submitted version.

## Funding

This study was supported, in part, by the Hubei Provincial Key Research and Development Program (No. 2020BCB008) and Clinical Research Pilot Project of Tongji Hospital (No. 2019YBKY026).

## Conflict of interest

The authors declare that the research was conducted in the absence of any commercial or financial relationships that could be construed as a potential conflict of interest.

## Publisher’s note

All claims expressed in this article are solely those of the authors and do not necessarily represent those of their affiliated organizations, or those of the publisher, the editors and the reviewers. Any product that may be evaluated in this article, or claim that may be made by its manufacturer, is not guaranteed or endorsed by the publisher.

## Supplementary material

Supplementary Figure 1 Validation of the nine hub genes expression in another external CD gene expression profile (GSE117993).

Supplementary Figure 2 Validation of the nine hub genes expression in external HSCR gene expression profile (GSE96854).

Supplementary Figure 3 The diagnostic value of the nine hub genes in another CD cohort (GSE126124).

Supplementary Figure 4 The histogram of the top 30 node in PPI network of common DEGs.
