# Supplementary figures and images for: Identification and validation of the common pathogenesis and hub biomarkers in Hirschsprung disease complicated with Crohn’s disease
Source: Front Immunol. 2022 Sep 28;13:961217. doi: 10.3389/fimmu.2022.961217 (PMC9555215; doi:10.3389/fimmu.2022.961217)

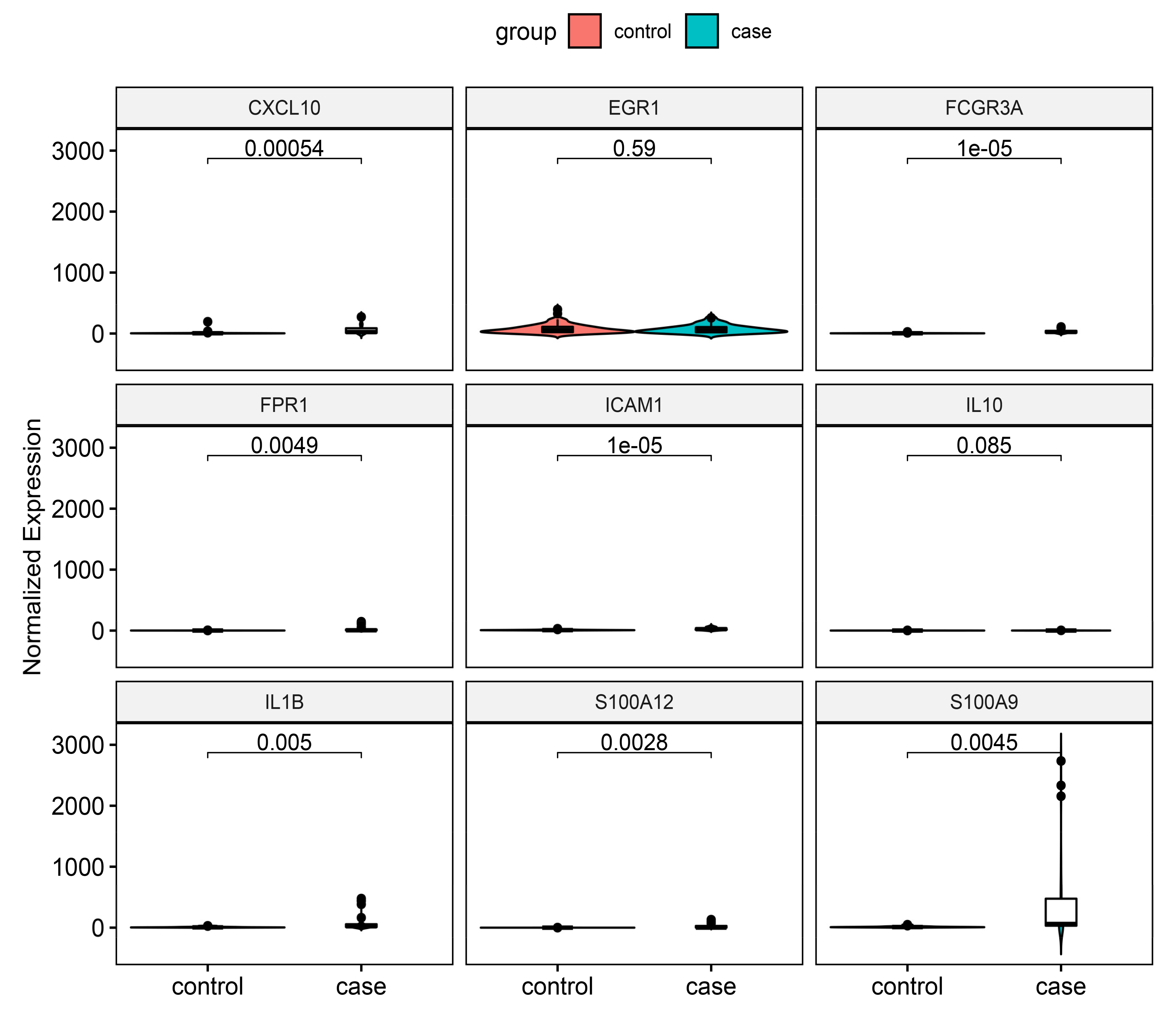

Supplement: Supplementary Figure 1 — Validation of the nine hub genes expression in another external CD gene expression profile (GSE117993). [file Image_1.tif]

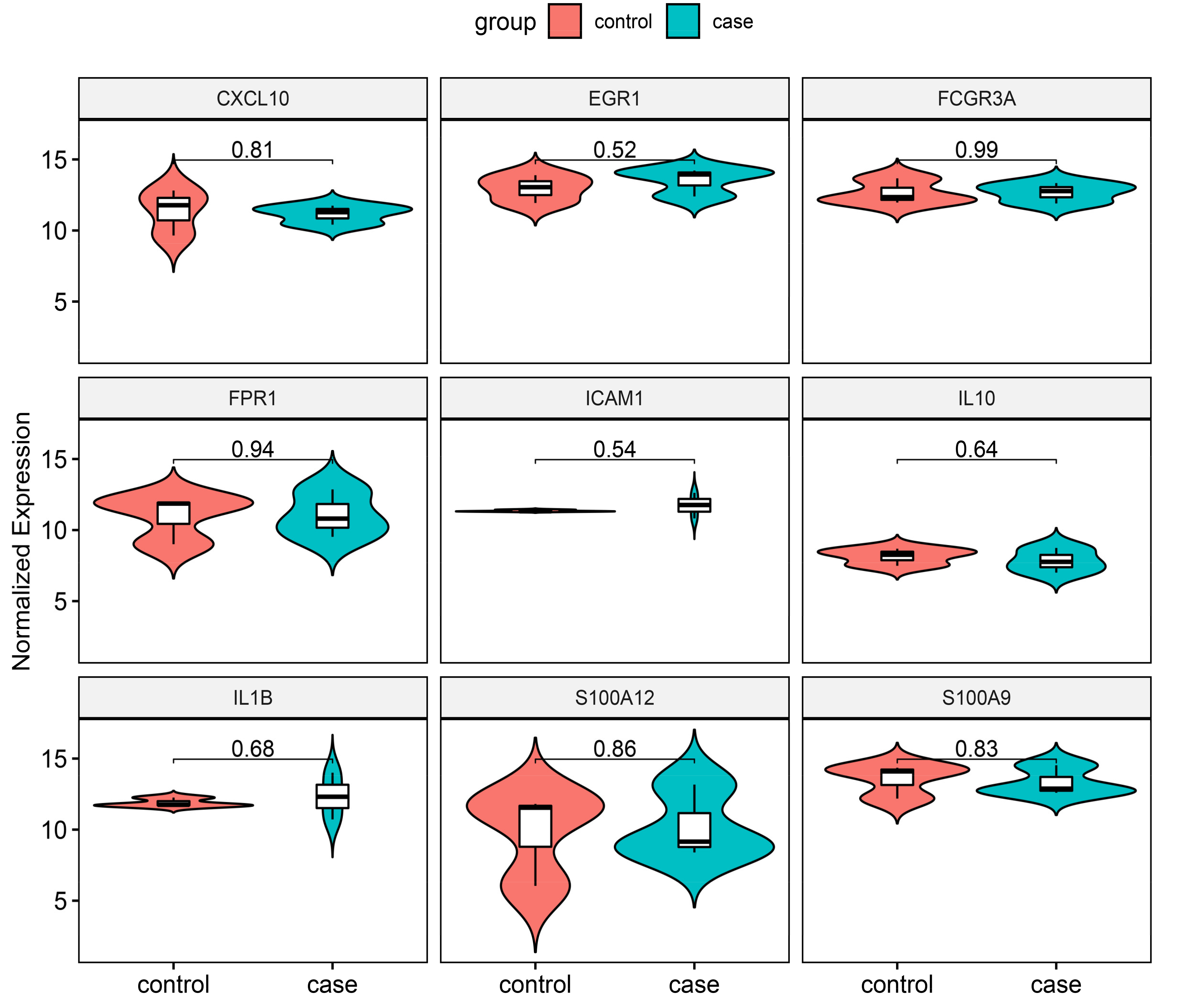

Supplement: Supplementary Figure 2 — Validation of the nine hub genes expression in external HSCR gene expression profile (GSE96854). [file Image_2.tif]

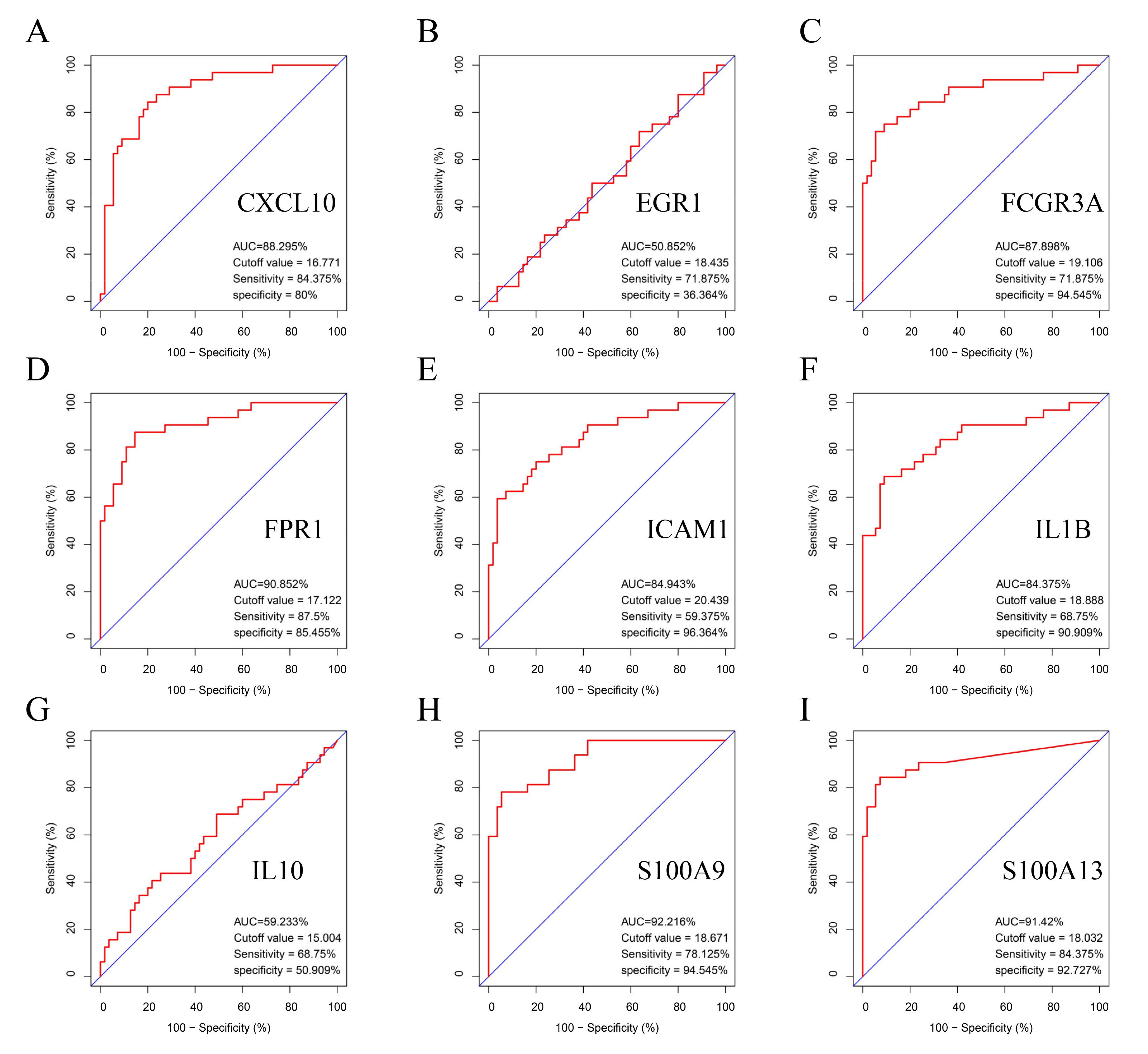

Supplement: Supplementary Figure 3 — The diagnostic value of the nine hub genes in another CD cohort (GSE126124). [file Image_3.tif]

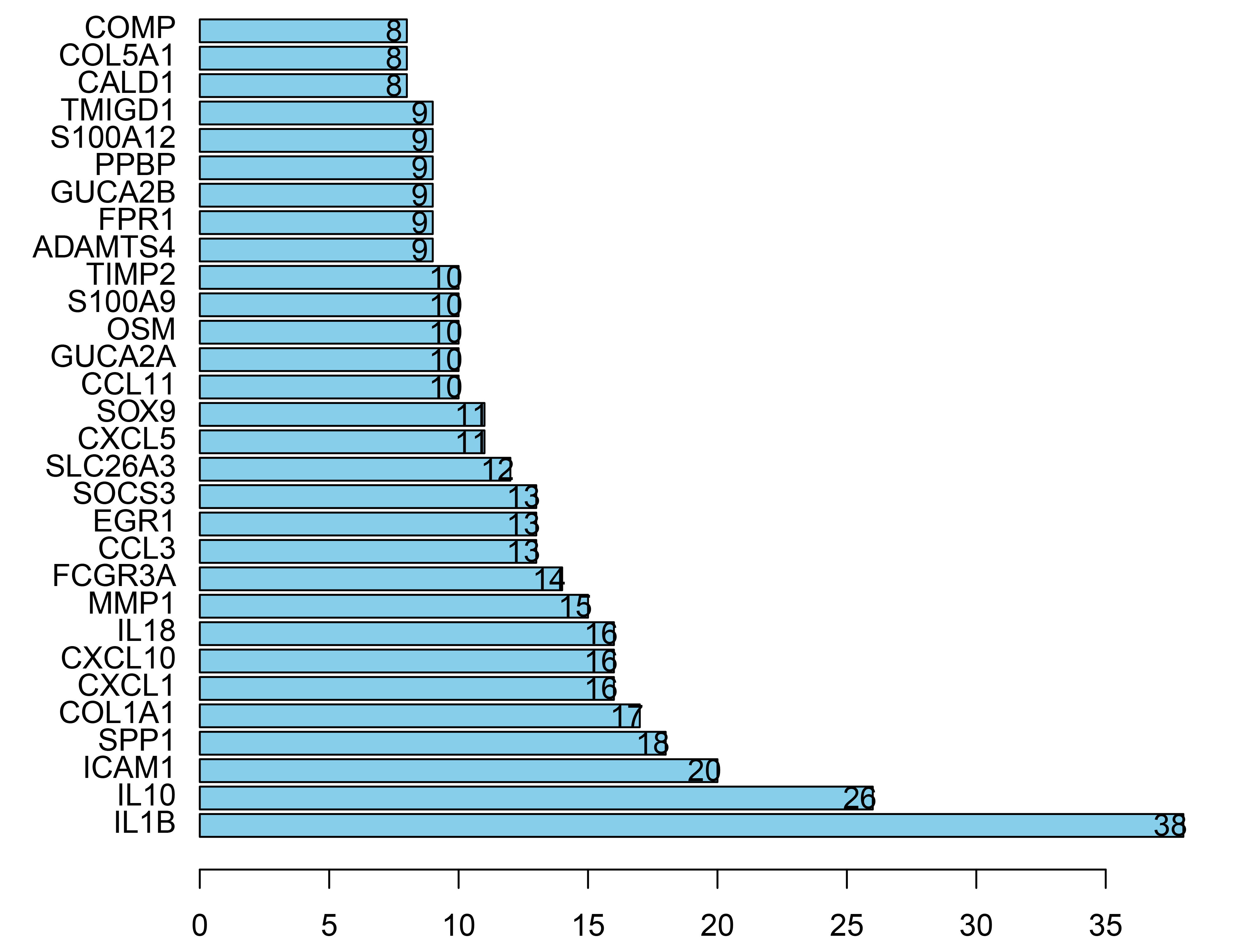

Supplement: Supplementary Figure 4 — The histogram of the top 30 node in PPI network of common DEGs. [file Image_4.tif]
